# Legislation for Midwives

**Published:** 1900-06-02

**Authors:** 


### The Institutional Workshop.

### LEGISLATION FOR MIDWIVES.

By a Correspondent.

### V.—CONCLUSION.

I pass from the consideration of the effect likely to be produced on midwives by the Bill now before Parliament to what is even more important—its effect on the mothers. Setting aside the obvious fact that whatever lowered the standard of midwifery would eventually work to the disadvantage of the mothers, it is, I think, beyond dispute that the condition of the poor in towns would be affected very favourably by the proposed measure.

The Bill would enable the practice of notoriously mischievous or careless women to be stopped altogether, and would act as a great protection to mothers not shrewd enough to find out and employ women of established reputation. In some parts of London, owing to the great demand for clinical material for teaching, any lying-in mother may have a bewildering variety of free "letters" to choose from, and there is hardly in any town so great a lack of trained nurses that the mother is forced to employ an unskilled person.

It is in remote country districts that the difficulties of registration begin. Any parish doctor whose district comprises widely-scattered hamlets and isolated cottages, knows that it is extremely difficult to get anyone at all to attend the mothers in their labour. In many districts such as these the district nurse does this work, receiving a small fee from her patient, but the amount paid to her in this way could never give her a living, and her salary must always be mainly defrayed by charitable contributions. Where there is no district nurse, there is generally in such localities one woman or another who has an elementary knowledge, gained from observation, of the duties required, and who will give her help, such as it is, if she is paid. It would, in my opinion, be equally injudicious to make such a woman into a registered midwife, or to render it penal for her to take money for attending her neighbour. To expect her to leave her own work and family at the cost of much personal inconvenience to undertake a serious office of this description, at the same time exposing her to a heavy fine, which in her case would certainly mean imprisonment, if she received a few shillings pay for exertions, is absurdly harsh on paper, and would be quite impracticable when translated into action. The real remedy for districts such as this lies in increased voluntary effort extending until there is no part of the kingdom so remote that the poor may not have the comfort of skilled nursing within their reach; but, meantime, we must beware how we frame the laws meant for their relief. I think the difficulty might possibly be met by conferring on the parish doctor or local supervising officer the power of licensing these amateur midwives to attend particular cases, when no regular midwife were within reach. If the Bill is to meet with cordial acceptance from those who have studied the circumstances of the poor in thinly populated parts of England, this point will need careful adjustment.

The third body of persons likely to be touched by the regulation of the midwives' calling are the members of the medical profession. There has been, and still is, in certain quarters, determined opposition to legislation on this subject, as likely to confer on midwives something of a professional status, and lead them to usurp the work of the medical practitioner. Dr. Cullingworth, in touching on this point, has very finely said, "As doctors we have no right to be considered, no right to exist, except so long as we serve the interests of the public." But, undoubtedly, the great body of general medical practitioners, who believe their interests to be in peril, are entitled to very special consideration at the hands of the law. They do more work for less pay than the members of any other profession, and their well-being is as important to the public as to themselves.

I have shown, however, that, so far from the midwives invading the province of doctors, it is the doctors who have intervened for the reform of an essentially feminine calling. When the medical profession had created the science of obstetrical surgery, there ensued a brief period when it was believed that the delivery of women in labour would pass once and for all into the sphere of the qualified doctor. This expectation was not justified by the event. The rate of mortality in child-birth steadily diminished as skill and learning replaced the barbarous methods of the ignorant midwife, and the advantage was reaped not only by rich mothers who could afford to pay the increased charge, but by the very poor who were attended gratuitously. Among that very large section of the community which may be described as the lower middle-class the services of the midwife have never, however, been superseded, and, as knowledge advanced, it was found that the safety of mother and child could be sufficiently safeguarded by a well-instructed nurse. There would be less opposition to the regulation of this ancient calling if this plain historical truth were realised by all concerned. If, after the lapse of over a century of opportunity on the part of medical practitioners, during which time they have had overwhelming advantages as regards knowledge, the proportion of mothers to-day who prefer the ministrations of their own sex is as high as 62 per cent., is it likely that midwives who are now more highly skilled, and of better social position than ever before, will become less in request? This question should have been fought out long ago when first the regular instruction of midwives began. It is their efficiency, not their registration, which will lead to the extension of their work, and to place them under supervision, while it will certainly tend to protect their employers, can in no degree extend their connection or injure the medical profession.

It may be thought, however, that the recognition of a higher order of midwives would be fraught with some danger, and that the more privileged "trained nurse midwives," for whom I have pleaded in a previous article, might arrogate to themselves offices which could not without risk to the patient be performed except under the direction of a medical man. I believe this fear to be altogether groundless. A woman, who has been taken out of some cottage home or from a manual occupation and subjected to three months' training only before being set to exercise these highly responsible duties, will very probably be imbued with an exalted notion of her own attainments, and can have very little idea of what constitutes danger for her patient, or of the very narrow limitations of her skill. It will be quite necessary that she should be watched and hedged round with restrictions. But the very essence of the three years' training which ought to be set as the desideratum for all midwives, is the recognition of what lies in their domain as distinguished from what lies outside it. These midwives will have worked in the presence of eminent surgeons, learnt to obey at a glance, to appreciate professional skill, and to be content with supremacy in their own special department. They have learnt enough to detect symptoms of danger, and to know at once when medical aid should be summoned. The higher the training and qualification demanded, the less fear will there be of any arbitrary action on the part of the midwife.

To conclude. The need for legislation has long been acknowledged, and the difficulties which have beset its advocates are lessening every day. I have tried to show that the proposals now before Parliament are lacking in one or two essential features, that the leading training schools for midwives should be accorded representation on the Central Board, that careful provision has yet to be made for the poorer mothers in thinly populated districts, and that the nurse-midwife who has obtained a three years' certificate of proficiency in medical and surgical nursing, in addition to her special knowledge of midwifery, should, for the honour of the calling, be granted some recognition on the part of the law above the mere "decent body" who has scraped through her L.O.S. examination after a three months' course.

The main principles of the Bill have met with cordial acceptance from nearly all, and if some still counsel delay it is rather than run any risk of marring by an imperfect system of registration the even more important work of education already most efficiently organised and thus permanently lowering the standard of midwifery in this country.

